# Comparison between Motor Performance of People with Multiple Sclerosis during a Virtual Reality Task Practiced on Concrete and Abstract Devices: A Cross-Sectional Randomized Study

**DOI:** 10.3390/brainsci14090916

**Published:** 2024-09-12

**Authors:** Camila Miliani Capelini, Giulianna Mendes Ferrero, Ana Maria Canzonieri, Roger Pereira Silva, Mauricio Ossamu Bando, Renata Martins Rosa, Cintia Ramari Ferreira, Talita Dias da Silva, Alessandro Hervaldo Nicolai Ré, Marcelo Massa, Luciano Vieira de Araújo, Fernando Henrique Magalhães, Carlos Bandeira de Mello Monteiro

**Affiliations:** 1 Graduate Program in Rehabilitation Sciences, Faculty of Medicine, University of São Paulo (FMUSP), São Paulo 01246-903, Brazilcarlosmonteiro@usp.br (C.B.d.M.M.); 2 Graduate Program in Physical Activity Sciences, School of Arts, Science and Humanities, University of São Paulo (EACH-USP), São Paulo 03828-000, Brazilalehnre@usp.br (A.H.N.R.); lvaraujo@usp.br (L.V.d.A.); 3 Brazilian Association of Multiple Sclerosis, São Paulo 04062-003, Brazil; 4 Graduate Program in Bioengineering, University Brazil, Sao Paulo 05508-010, Brazil; 5 Department of Physical Therapy, Faculty of Sciences and Technology (FCT/UNESP), State University of São Paulo, Presidente Prudente 14884-900, Brazil

**Keywords:** multiple sclerosis, virtual reality exposure therapy, learning, motor activity

## Abstract

Multiple sclerosis (MS) is an autoimmune demyelinating disease of the central nervous system with unknown etiology, resulting in various impairments that necessitate continuous rehabilitation to enhance functionality, quality of life, and motor function, including through Virtual Reality (VR) therapy. Comparing tasks in virtual environments and their potential skill transfer to real-world settings could aid in optimizing treatment programs to improve motor performance in individuals with MS. This study aimed to determine whether practicing acquisition and retention phases using two distinct interfaces (concrete—Touch Screen or abstract—Kinect system) affects performance in a subsequent task using a different interface (transfer phase). A randomized clinical trial was conducted with 56 volunteers with MS and 41 controls. Participants engaged in a computer game where they burst as many bubbles as possible within 10 s per attempt. After the acquisition and retention phases, all participants switched interfaces (e.g., those using Kinect switched to Touchscreen and vice versa). Significant performance improvements were observed in both groups during the acquisition phase, which were maintained in the retention phase. Although the abstract interface was more challenging for both groups, only the MS group that practiced with the abstract interface successfully transferred their improvements to the concrete interface. Thus, despite the increased difficulty of the abstract task during practice, it led to better performance transfer when required to complete a subsequent concrete task, suggesting that abstract devices may be beneficial in clinical practice for improving motor function in people with MS.

## 1. Introduction

Multiple sclerosis (MS) is a progressive neurodegenerative disease of the central nervous system (CNS) [1], characterized by symptoms such as muscle weakness, difficulties in locomotion, and alterations in muscle tone [2,3,4]. Consequently, various interventions aim to improve function, including medications and continuous rehabilitation programs [4]. One intervention that has gained traction for people with physical disabilities is Virtual Reality (VR) [5,6,7].

The effects of VR interventions for people with MS have been studied, revealing positive outcomes in motivation [4], walking [8], and balance [9,10]. A systematic review by Casuso-Holgado et al. (2018) [11] on the effectiveness of VR training for gait rehabilitation in MS showed positive results. Furthermore, a review by Massetti et al. (2016) [12] concluded that VR is a viable alternative to traditional rehabilitation, aiding in the improvement of motor and cognitive deficits. Recently a systematic review and meta-analysis highlights the potential of VR-based rehabilitation to enhance cognitive function and mood in people with MS, suggesting it could be an effective treatment option in the future [13].

Despite existing studies on the use of VR in MS, there remains a gap in the literature regarding the comparison of performance between abstract tasks (without physical contact) and concrete tasks (with physical contact). Monteiro et al. (2013) [14] noted that in abstract environments, characteristic of VR tasks, participants simulate task performance, resulting in a different spatio-temporal organization of movement compared to natural (concrete) environments, particularly among individuals with movement disorders. For instance, using a device like the Kinect, which involves no physical contact, provides abstract information and may result in different performance outcomes compared to the same task performed with a Touch Screen, which offers more concrete information through physical contact [14,15,16]. Tactile feedback in concrete tasks includes sensations of touch, temperature, and surface friction [17], which can positively influence performance.

Considering the above deliberation, we conducted this study to evaluate individuals with MS and a control group of typically developing individuals during the performance of similar tasks with two different forms of interaction (interface devices) in a motor learning protocol. One device involved movements without touching the screen (abstract task), while the other required direct contact (concrete task).

The primary objective was to determine if there are performance differences when the same task is performed using different interfaces: abstract or concrete. Additionally, our protocol aimed to establish whether the acquisition and retention phases with a specific interface influence subsequent performance with a different interface (transfer phase). We hypothesized that, in all protocols, the control group would outperform the MS group. Furthermore, we anticipated that the task performed with the abstract interface would pose greater difficulty during acquisition and retention for both groups, but would result in better performance during the transfer to the concrete interface.

## 2. Materials and Methods

This study was a randomized clinical trial conducted at ABEM—the Brazilian Multiple Sclerosis Association, located in São Paulo, SP. Fifty-six volunteers diagnosed with multiple sclerosis (MS), confirmed by a specialist and through clinical and neuroimaging exams, participated in the study, along with 41 volunteers without MS, who comprised the control group (CG). All participants selected for the study signed an informed consent form, which was previously approved by the Research Ethics Committee of the Faculty of Medicine of the University of São Paulo (CAAE: 89788518.5.0000.0065, approval date: 21 June 2018).

The inclusion criteria for participants with MS were: a diagnosis of Multiple Sclerosis, an age range between 20 and 60 years, an Expanded Disability Status Scale (EDSS) score between 0 and 8, and the absence of an outbreak in the previous two months. The exclusion criteria included difficulty playing computer games due to motor limitations (fatigue, deformity, or muscle weakness in the upper limbs that prevent wave movement) and visual impairments (optic neuritis and diplopia).

### 2.1. Randomization

After the recruitment process, the volunteers were included in the protocol following the inclusion criteria. Next, the terms of consent and assent were presented to participants as well as the procedures and objectives of the study.

Subsequently, the participants from both MS and Control Group were randomly allocated into two subgroups (Kinect and Touchscreen) with a 1:1 ratio defined by website (randomization.com) by an independent researcher who was not involved with the recruitment of participants nor the evaluations, with the aim of keeping the process as a blind distribution.

- Kinect Interface (Ki)—Abstract Task: Participants used a wave movement to complete the task during the acquisition and retention phases, followed by a transfer to the Touchscreen interface.
- Touchscreen Interface (Ts)—Concrete Task: Participants used hand movements to touch the screen and complete the task during the acquisition and retention phases, followed by a transfer to the Kinect system interface.

### 2.2. Assessment Scales

The Expanded Disability Status Scale (EDSS) [18] was used to classify MS-related disability, along with various physical assessments, including the Modified Fatigue Impact Scale (MFIS) to assess fatigue [19], the Box and Block test to assess manual dexterity [20], and Functional Reach to evaluate individual stability [21,22,23]. Additionally, the Divided and Alternating Attention Test (TEADI and TEALT) was used for psychological assessment, measuring the ability to divide attention, i.e., the individual’s capacity to respond to more than two stimuli simultaneously during the task [24].

### 2.3. Task Description

**Figure 1:**
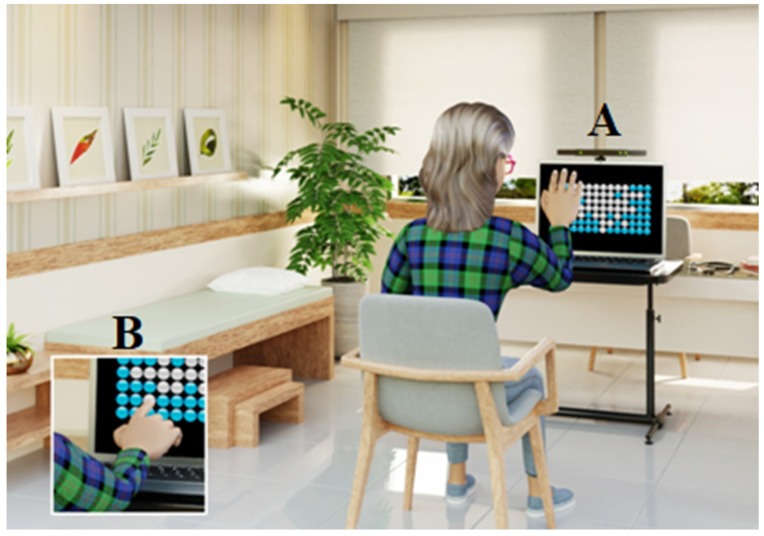
Representation of a participant with Multiple Sclerosis performing the Reaching Bubbles task using the Kinect system (**A**); (**B**) participant using a touchscreen device.

The computer game titled Reaching Bubbles (developed by the Information Systems group of the School of Arts, Sciences, and Humanities, EACH-USP) was used. The task presented 126 bubbles on the computer screen, arranged in lines and columns (Figure 1). The objective was to burst as many bubbles as possible within 10 s (per attempt) until a total of 300 bubbles was reached.

### 2.4. Protocol

A short-term motor learning protocol was employed to explore whether tasks performed in a virtual environment can transfer to the real world and vice versa, including acquisition, retention, and transfer phases, as demonstrated in studies by Monteiro et al. (2014), Silva et al. (2020), and Monteiro et al. (2017) [14,25,26]. Individuals with MS and a control group (typically developing individuals) were assessed while performing similar tasks using two different interface devices. One group used the Kinect system with wave movements (abstract) while the other group used a Touch Screen with finger contact (concrete) and, after these phases, all participants switched interfaces for the transfer phase, with those who used Kinect moving to Touch Screen and vice versa.

**Figure 2:**
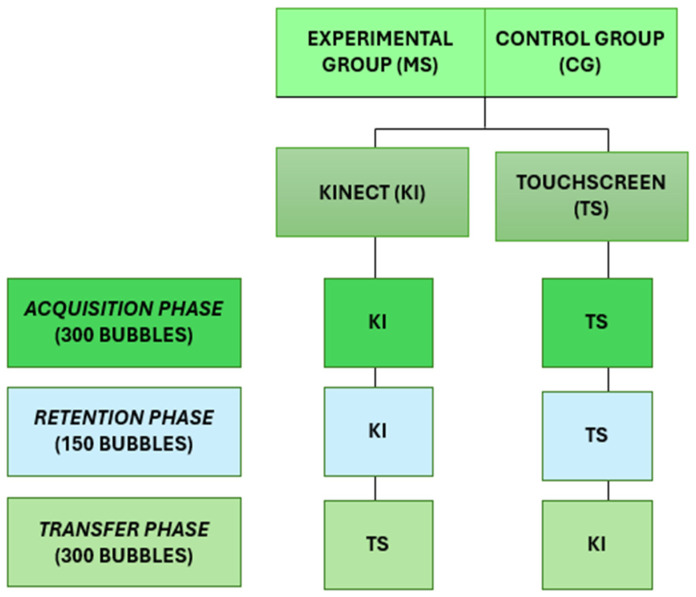
Study design.

Participants performed the Reaching Bubbles game for as many attempts as necessary to reach 300 bubbles in the acquisition phase. After a 10-min rest period, participants moved to the retention phase, aiming to burst 150 bubbles. At the end of the protocol, participants were required to burst 150 bubbles in the transfer phase (using a different device). Figure 2 represents the study design.

### 2.5. Data Analysis

Participant characteristics (age, sex, type of MS, time since diagnosis) and clinical evaluations using functional scales were analyzed through descriptive statistics. A one-way ANOVA was conducted to determine whether there were significant differences in the mean scores on the functional scale and in age across the interface groups (Kinect (Ki) and Touchscreen (Ts)). For comparisons between groups, interfaces, and attempts, the dependent variable used was the number of bubbles burst. The dependent variables were analyzed using a 2 (group: MS, CG) × 2 (Interfaces: Kinect, Touchscreen) × 2 (Attempt) ANOVA with repeated measures on the last factor. For the Attempt factor, separate comparisons were made for acquisition (first acquisition attempt—FA versus last acquisition attempt—LA), retention (LA versus retention attempt—R), and transfer (LA versus transfer attempt—T). Post hoc comparisons were performed using the Tukey–HSD test (*p* < 0.05).

## 3. Results

A total of 97 individuals participated in this study, with 56 in the MS group and 41 in the control group (CG). Table 1 provides the characterization of the sample within the groups and subgroups (Kinect—Ki and Touchscreen—Ts), according to sex, age (mean and standard deviation), functional and cognitive tests, time since diagnosis of the disease and previous experience with computer and games. There were significant differences between MS and Control groups regarding Box and Block test, functional reaching—anterior and lateral left. No significant difference between and subgroups of interfaces were found. The tests TEALT and TEADI were classified as “Superior: 1; Average Superior: 2; Average: 3; Average Inferior: 4; Inferior: 5” (Table 1).

**Figure 3:**
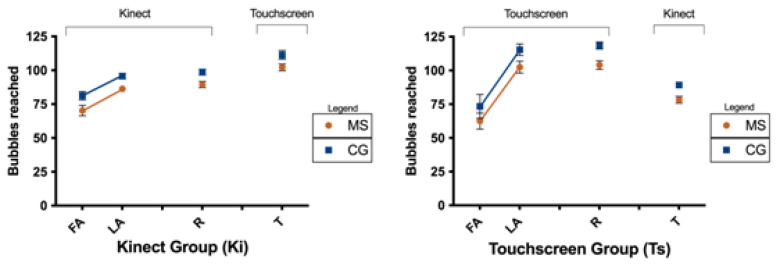
Number of Bubbles Burst by the Groups During the Stages of Motor Learning. Abbreviations: FA: First Attempt of Acquisition Phase; LA: Last Attempt of Acquisition Phase; R: Retention; T: Transfer; CG: Control Group; MS: Multiple Sclerosis Group.

The performance results in the virtual game Reaching Bubbles during each motor learning phase are shown in Figure 3. The number of bubbles burst in the first attempt (FA) and last attempt (LA) of the acquisition phase reflects the total number of bubbles burst in a single attempt (10 s), within the protocol of 300 bubbles in the acquisition phase.

### 3.1. Acquisition

Significant effects were found for Attempt, F(1, 89) = 85.3, *p* < 0.001, ŋ_p_^2^ = 0.49, and Group, F(1, 89) = 5.2, *p* = 0.026, ŋ^2^ = 0.06. These results indicate that participants increased the number of bubbles burst from the First Attempt (FA) (M = 72 ± 3) to the Last Attempt (LA) (M = 100 ± 3). Moreover, the MS group demonstrated worse performance (M = 82 ± 3) compared to the CG (M = 90 ± 2). Although there was no significant interaction, the post hoc test revealed that the difference between the MS and CG occurred only in the Last Attempt (M = 95 ± 3 and 105 ± 3, respectively).

An interaction was observed for Attempt by Interface, F(1, 89) = 14.7, *p* < 0.001, ŋ_p_^2^ = 0.14. The post hoc test showed that individuals performed better with the touchscreen (M = 109 ± 3) than with the Kinect (M = 91 ± 3), but only in the Last Attempt. The results are presented in Figure 3.

### 3.2. Retention

There was no significant difference between the Last Attempt and Retention trial in either group or interface. This result demonstrates that the participants retained the ability acquired during the acquisition phase.

Significant effects were found for Group, F(1, 88) = 13.2, *p* < 0.001, ŋ_p_^2^ = 0.13) and Interface, F(1, 88) = 31.6, *p* < 0.001, ŋ_p_^2^ = 0.27. These results suggest that the control group showed better performance (M = 106 ± 3) when compared to the MS group (M = 96 ± 2) and burst a larger number of bubbles with the touch screen interface (M = 109 ± 3) when compared to Kinect (M = 93 ± 3).

### 3.3. Transfer

Significant effects were found for Attempt, F(1, 87) = 9.4, *p* = 0.003, ŋ_p_^2^ = 0.10, and Group, F(1, 87) = 15.5, *p* < 0.001, ŋ_p_^2^ = 0.15. These results suggest that participants decreased the number of bubbles burst from Retention (R) (M = 100 ± 2) to Transfer (T) (M = 95 ± 2). Additionally, the control group burst a greater number of bubbles (M = 103 ± 3) than the MS group (M = 92 ± 2). Although there was no interaction between Attempt and Interface, the post hoc test revealed that the group which performed the acquisition and retention phases on the touchscreen (M = 109 ± 3) showed decreased performance when transferring to the Kinect (M = 83 ± 2). Conversely, the group that performed acquisition and retention on the Kinect (M = 91 ± 3) exhibited improved performance when transferring to the touchscreen (M = 106 ± 2).

To further assess whether performance in the transfer phase was better than in the first trial for both groups and sequences, we conducted separate comparisons between the first attempt (FA) of acquisition and Transfer (T) for each group and sequence. An independent samples t-test indicated a significant difference for the MS group between the FA of the Ts Group and T of the Ki Group on the touchscreen (*p* < 0.001), but no significant difference was found between the FA of the Ki Group and T of the Ts Group on the Kinect. For the CG, significant differences were observed for both conditions (*p* = 0.040 and *p* < 0.001, respectively).

### 3.4. Correlation Analysis

A correlation analysis considering attempts LA, FA, R, and T, as well as the improvement in the number of bubbles burst from the first to the final practice blocks (difference LA—FA), was performed to identify which factors (EDSS; age; anterior, right, and left functional reach; alternate attention test—TEALT; divided attention test—TEADI; manual dexterity test—Box and Block; modified fatigue impact scale—MFIS) were correlated with the degree of learning during practice for the MS group.

The results showed that the older the participant, the lower was their performance in the first and last block of attempt at the acquisition phase of the game. The EDSS pointed out that, the more severe the impairment from MS, the worse the performance in the last block of acquisition and in the retention phase. The tests TEALT and TEADI were classified as “Superior: 1; Average Superior: 2; Average: 3; Average Inferior: 4; Inferior: 5”, and the results showed that, the lower the classification, the worse the performance in the task. Regarding functional reach and Box and Block test, those positively correlated mainly with transfer of the task, in which, the higher the functional reach and the score in the Box and Block, the better the performance in the transfer phase of the task. Lastly, the higher the fatigue impact (MFIS), the worse the performance in the transfer test. These results are presented in Table 2.

## 4. Discussion

Considering the results, our hypothesis was partially confirmed. The control group demonstrated better performance throughout most of the protocol, and the task performed with the Kinect system presented greater difficulty in both acquisition and retention for both groups, with better transfer performance to the touchscreen. However, this improvement in performance during the transfer phase was only observed in the MS group. These results will be discussed in detail below.

### 4.1. Comparison between Groups (MS and Control Group)

The results indicated that in both interfaces (Kinect—Ki and Touchscreen—Ts), there was an improvement in game performance for both groups (MS and CG), as evidenced by the increase in the number of bubbles burst from the first to the last attempt of the acquisition phase. Moreover, during the retention phase, there was no difference between performance in the last acquisition attempt (LA) and retention (R), suggesting that participants maintained the skill acquired during the acquisition phase across both interfaces (Ki and Ts).

As hypothesized, the CG outperformed the MS group in most protocol phases, meaning that individuals without MS burst more bubbles in the game than those with MS. Leocani et al. (2007) [28] corroborate these findings, having used a task that required tracking a target object projected on a screen, and demonstrated that the MS group’s performance was inferior to that of the control group. Dana et al. (2019) [29] also found a lack of precision during a computerized visual stimulus with a manual response applied to people with MS, further showing that motor deficits were more pronounced in the MS group compared to the CG. Additionally, our findings revealed a correlation between MS assessment scales and game performance, indicating that higher age and lower scores in various functional tests (e.g., box and block test, functional reach, and attention) were associated with poorer task performance.

These results align with existing literature, which suggests that motor impairments in MS, such as muscle weakness [30], fatigue [3,31], and spasticity [32], as well as difficulties in executive functions like attention, memory, concentration, and processing speed [2], could account for the poorer performance in the MS group. Moreover, the inconsistency in performance and inaccuracy in executing motor tasks that require time synchronization may be related to changes in the central nervous system of individuals with MS, resulting in slower and delayed responses that affect task effectiveness [29]. In this context, Guimarães et al. (2012) [33] and Binétruy et al. (2016) [34] observed that people with MS experience deficits in information processing speed, mainly due to reduced nerve conduction speed secondary to demyelinating lesions.

### 4.2. Comparison between Interfaces (Kinect and Touchscreen)

Regarding the different interfaces, our results showed that the concrete task (touchscreen) led to better performance, with both groups bursting more bubbles in the touchscreen interface compared to Kinect during the acquisition and retention phases. This improvement is likely to be due to the use of tactile sensations characteristic of touchscreens, which enhances the sensitivity to stimuli and provides a more efficient communication channel, leading to better performance [35,36].

Despite better performance in the concrete task, we observed that both groups practicing the touchscreen task during acquisition and retention showed worse performance when transferring to Kinect. However, both groups that practiced acquisition and retention with the Kinect system showed improved performance when transferring to the touchscreen interface. This transfer of performance is evident when comparing the retention and transfer phases for both groups. Notably, the influence of practicing an abstract task first was more pronounced in the MS group. Specifically, individuals with MS who began practice with Kinect (abstract first) demonstrated better subsequent performance on the touchscreen compared to those with MS who started practice on the touchscreen (i.e., practice with Kinect led to an increase in performance during the touchscreen transfer, with a higher value compared to the group with MS that started on the touchscreen).

This finding is the most significant result of our study and could be considered a preliminary step toward indicating that practicing an abstract task may improve performance in a subsequent concrete task for individuals with MS. The Kinect system, being relatively abstract with intangible objects, may present a more challenging task (as evidenced by both groups’ poorer performance during Kinect practice). This difficulty during an abstract task could facilitate sensorimotor adaptation and lead to better performance in a subsequent concrete task.

This conclusion is supported by studies comparing the performance of individuals with altered posture and movement in two tasks differing in degrees of abstraction (concrete vs. abstract). Freitas et al. (2019) [15] and Massetti et al. (2018) [12] (Duchenne muscular dystrophy), Moraes et al. (2020) [37] (Autism spectrum disorder), Leal et al. (2020) [38] and Monteiro et al. (2014) [14] (Cerebral palsy), and Trevizan et al. (2018) [39] (Amyotrophic lateral sclerosis) found similar results, where individuals with disabilities demonstrated performance transfer to a concrete task after practicing a similar task on a more challenging abstract device, such as Kinect or a webcam. These findings support the generalization of this effect to other populations with neurological disorders, suggesting that VR abstract devices could enhance functional abilities in real-life tasks. Furthermore, these results encourage the development of new research protocols for other neurological disorders.

### 4.3. Limitations and Future Studies

While our study yielded interesting results regarding the use of VR for individuals with MS, several limitations should be noted: (1) the lack of familiarity with devices without physical contact could have influenced the results; (2) our findings may not be generalizable to other VR tasks, so future studies are needed to reinforce our findings; (3) we used a non-immersive VR task but, with the growing adoption of immersive VR technologies, such as VR headsets, future studies should compare abstract and concrete tasks in these fully immersive environments. Exploring these advanced environments could significantly enhance our understanding of how different VR settings impact adaptation and learning processes; (4) our study yielded interesting results using a VR task to assess motor performance in people with MS. However, we did not examine the potential effects and influence of cognitive function and mood, which could have provided valuable insights. Future research should explore these aspects to gain a more comprehensive understanding, and (5) we did not analyze movement patterns during task execution, a critical aspect that could provide valuable insights into how individuals adapt and develop compensation strategies. Understanding these patterns is essential for optimizing rehabilitation approaches. Therefore, we recommend that future studies include kinematic assessments to capture these important dynamics.

## 5. Conclusions and Clinical Applications

Based on our results, both groups (MS and CG) showed improved performance regardless of the device used (concrete or abstract), although people with multiple sclerosis performed worse than the control group across all protocols. However, the most significant result for clinical application was observed in the transfer phase, where practice with the Kinect system (abstract) showed a positive transfer to the touchscreen (concrete) only in the MS group. Thus, the implementation of an VR task that requires abstract movements, i.e., without touch, can be considered a promising approach in clinical practice, as it might enhance performance in individuals with MS when they need to transfer the acquired abilities to real-life tasks. Free access options, such as the ones available at www.movehero.com.br/en, accessed on 15 October 2024 and https://paterland.com/en-GB.htm, accessed on 15 October 2024, would be of great benefit for people with MS as well as other populations of people with neurological disorders.

## Figures and Tables

**Table 1:** Characterization of the Sample by Subgroups.

	MS Group		Control Group	
	Ki	Ts	Ki	Ts
n	26	30	21	20
Sex (n)				
- Female	19	19	14	12
- Male	7	11	7	8
Age (mean ± SD)	44.65 ± 10.43	44.03 ± 10.87	41.05 ± 10.81	40.89 ± 11.82
Box and blocks (mean ±SD)	37.9 ± 9.5	35.7 ± 9.4 *	42.6 ± 4.8	41.7 ± 6.6 *
Functional reaching—anterior (mean ± SD)	34.7 ± 8.9	30.0 ± 7.9 *	33.9 ± 6.3	37.3 ± 5.1 *
Functional reaching—lateral right (mean ± SD)	25.6 ± 7.4	24.0 ± 6.7	27.8 ± 5.7	26.8 ± 5.2
Functional reaching—lateral left (mean ±SD)	25.3 ± 6.0	22.3 ± 6.5 *	25.7 ± 5.0	26.6 ± 3.7 *
EDSS (mean ±SD)	3.5 ± 2.1	3.6 ± 2.3	-	-
MFIS (mean ±SD)	29.6 ± 14.2	34.8 ± 19.5	-	-
TEADI (n)			-	-
1	3	1		
2	4	7		
3	0	0		
4	8	3		
5	11	14		
TEALT (n)			-	-
1	3	2		
2	3	4		
3	0	1		
4	7	8		
5	13	10		
Time since diagnosis (n)			-	-
Up to 1 year	3	1		
From 1 to 3 years	1	4		
From 3 to 5 years	4	3		
From 5 to 10 years	10	6		
Over 10 years	8	15		
No information		1		
Experience with computer (n)				
Yes	25	23	17	17
No	3	4	0	1
Experience with games (n)				
Yes	14	8	10	5
No	14	19	7	12

Abbreviations: MS = Multiple Sclerosis; Ki = Kinect; Ts = Touchscreen; n = sample size; SD = Standard Deviation); alternate attention test—TEALT; divided attention test—TEADI. * means *p*-value < 0.05 between MS and Control Groups.

**Table 2:** Correlation analysis. Adapted from [27].

		Age	EDSS	TEALT	TEADI	Anterior Functional Reach	Lateral Functional Reach (Right)	Lateral Functional Reach (Left)	Box Block Test	MFIS
Diff	r	0.086	−0.19	−0.063	−0.002	−0.074	−0.061	−0.264	−0.214	−0.075
	*p*-value	0.541	0.181	0.668	0.991	0.6	0.664	0.056	0.124	0.595
FA	r	−0.369 **	−0.168	−0.24	−0.316 *	0.237	0.252	0.360 **	0.385 **	0.001
	*p*-value	0.006	0.239	0.096	0.027	0.087	0.068	0.008	0.004	0.997
LA	r	−0.350 *	−0.464 **	−0.376 **	−0.387 **	0.2	0.235	0.1	0.199	−0.099
	*p*-value	0.01	0.001	0.008	0.006	0.151	0.09	0.475	0.153	0.483
Retention	r	−0.267	−0.512 **	−0.509 **	−0.397 **	0.246	0.372 **	0.244	0.268	−0.117
	*p*-value	0.056	0	0	0.005	0.079	0.007	0.082	0.054	0.408
Transfer	r	−0.119	−0.249	−0.191	−0.353 *	0.502 **	0.295 *	0.385 **	0.389 **	−0.290 *
	*p*-value	0.4	0.082	0.194	0.014	0	0.034	0.005	0.004	0.037

Abbreviations: Diff: difference from first to last attempt of acquisition phase; FA: first attempt of acquisition phase; LA: last attempt of acquisition phase; TEALT: alternate attention test; TEADI: divided attention test; MFIS: modified fatigue impact scale. An asterisk (*) indicates *p* < 0.05, while two asterisks (**) indicates *p* < 0.005.

## Data Availability

The raw data supporting the conclusions of this article will be made available by the authors on request.
